# Global burden and trends of disability‐adjusted life years and mortality for decubitus ulcer: A systematic analysis

**DOI:** 10.1111/iwj.14604

**Published:** 2024-02-01

**Authors:** Hongfan Ding, Zhenghao Yu, Hongwu Yao, Xiao Xu, Yunxi Liu, Minliang Chen

**Affiliations:** ^1^ Senior Department of Burns and Plastic Surgery The Fourth Medical Center of PLA General Hospital Beijing China; ^2^ Department of Infection Management and Disease Control The First Medical Center of PLA General Hospital Beijing China

**Keywords:** decubitus ulcer, disability‐adjusted life years, global burden of disease, mortality

## Abstract

The management of chronic wounds has presented a significant dilemma, which is evident not only in clinical treatment but also in the substantial burden it places on medical resources. The global COVID‐19 pandemic in 2020 is likely to further exacerbate this trend. Therefore, it is imperative to delve deeper into the impact of chronic wound on disease burden across different regions and populations. In this study, we focused on decubitus ulcers (DU) as representative chronic wounds and utilized data from the Global Burden of Disease (GBD) 2019 database (http://ghdx.healthdata.org/gbd-results-tool) pertaining to age, gender, region, year and socio‐demographic index (SDI) group. Disability‐adjusted life years (DALYs) and mortality were utilized as indicators to assess the burden of DU. The analysis and visualization were performed using R software (version 4.2.3). A decrease in the global ASRs of DALYs and mortality for DU was observed across most regions between 1990 and 2019. The reduction in burden was particularly significant in regions characterized by a high SDI, while regions with a high‐middle SDI experienced an increase. The burden of DU increased with age for both males and females, with males generally experiencing a higher burden compared to females. Strengthening population‐based data on the prevalence of DU and implementing dynamic monitoring at the public health level will enable policymakers to develop evidence‐based strategies for efficient allocation of healthcare resources.

## 1 INTRODUCTION

The management of chronic wounds has always posed a significant challenge and burden for the social medical system, particularly in light of the ageing society.^1^ Considering chronic wounds as a complex syndrome necessitating multidisciplinary intervention is justifiable due to its high prevalence, multi‐factorial nature and significant impact on morbidity and mortality.^2^ Given the incidence and cost implications associated with chronic wounds, there is an imperative not only to enhance prevention, early diagnosis and treatment but also to elucidate their prevalence and impact on healthcare systems. This will ensure appropriate allocation of resources towards disease management and prevention.

The prevalence of pressure injury among hospitalized patients in the United States is estimated to range from 5% to 15%, with an annual treatment cost for these injuries as high as $17.8 billion.^3^ However, no assessment has been reported regarding changes in DALYs and mortality rates associated with DU across different regions, countries, ages and genders. In this study, we utilized the 2019 GBD reports on DU to analyse data based on gender, age groups and SDI. The aim was to provide comprehensive information on DALYs and mortality rates within specific regions for informed decision‐making by healthcare professionals in resource allocation and cost reduction strategies related to DU.

## 2 METHOD

### 2.1 Data source

In this study, we collected data from the GBD 2019 database (http://ghdx.healthdata.org/gbd-results-tool), which was developed and published by the Institute for Health Metrics and Evaluation at the University of Washington. The GBD study 2019 assessed different diseases and injuries in 204 countries and regions from 1990 to 2019 and provided comparable data for the public to estimate the burden of different diseases.^4^

The publicly available data of DU were collected from the GBD 2019 database. We extracted data from the GBD 2019 database separately in various classifications of age, gender, region, SDI and year. The categorization of age groups and region followed the classification of the GBD 2019 database, in which people younger than 1 year were excluded. The disease burden data of DU for each year from 1990 through 2019 are presented separately by both‐sex, male and female.

The SDI was divided into five categories according to previous literature: low, low‐middle, middle, high‐middle and high SDI.^5^, ^6^ The SDI is a comprehensive indicator of national and regional development status, which is expressed from 0 to 1, and a region with a higher SDI indicates a better development status related to health outcomes.^7^ The SDI values for all regions were available from the GBD 2019 study. Following the classification described above, the number and ASR on DALYs and mortality for DU were selected as indicators for the evaluation of disease burden.

### 2.2 Disease burden

The disease burden of DU was estimated by DALYs and deaths across age, gender, region and SDI, which were presented in absolute number and ASR with 95% uncertainty interval (UI). The ASR was calculated on the basis of the World Health Organization World Standard Population Distribution, which made it possible to compare differences between groups of different age demographics. DALYs is a metric defined as the total healthy life years lost from morbidity to death, which is calculated as the sum of years of life lived with disability (YLDs) and years of life lost (YLLs). All data of disease burden, including number and ASR of DALYs and deaths, were obtained from the GBD Results Tool (http://ghdx.healthdata.org/gbd-results-tool).

### 2.3 Data analysis

The absolute number and ASR (per 100 000 population) along with their 95% UI were used to illustrate the burden of the DALYs and deaths for DU, and the DALYs and deaths were compared across different sex, age, SDI and location. The estimated annual percentage change (EAPC) and its 95% confidence interval (CI) were used to indicated the temporal trend from 1990 to 2019. EAPC was an indicator that describes the trend in the ASR, which was used to assess the trend in the ASR of DU from 1990 to 2019. A regression line was fitted by converting the ASR to logarithmic form, namely: y = α + βx + ɛ, where y = ln (ASR) and x = year. EAPC was calculated as 100 × (exp(β)‐1), and its 95% CI can be obtained from the regression line.^8^

The ASR demonstrates an upward trend when both the EAPC and the lower boundary of the 95% CI exceed 0. In contrast, the ASR exhibits a downward trend when both the EAPC and the upper boundary of the 95% CI are less than 0. Otherwise, the ASR is considered stable if the 95% CI of the ASR includes 0. The heat map was used to depict the global distribution of DALYs and death rate in different age groups for DU, and the global map was drawn to describe the ASR of DALYs and deaths across different regions (R package: ggplot2, pheatmap). The analysis and visualization were performed by R software (version 4.2.3).

## 3 RESULTS

### 3.1 Global burden and changes for DU

**FIGURE 1:**
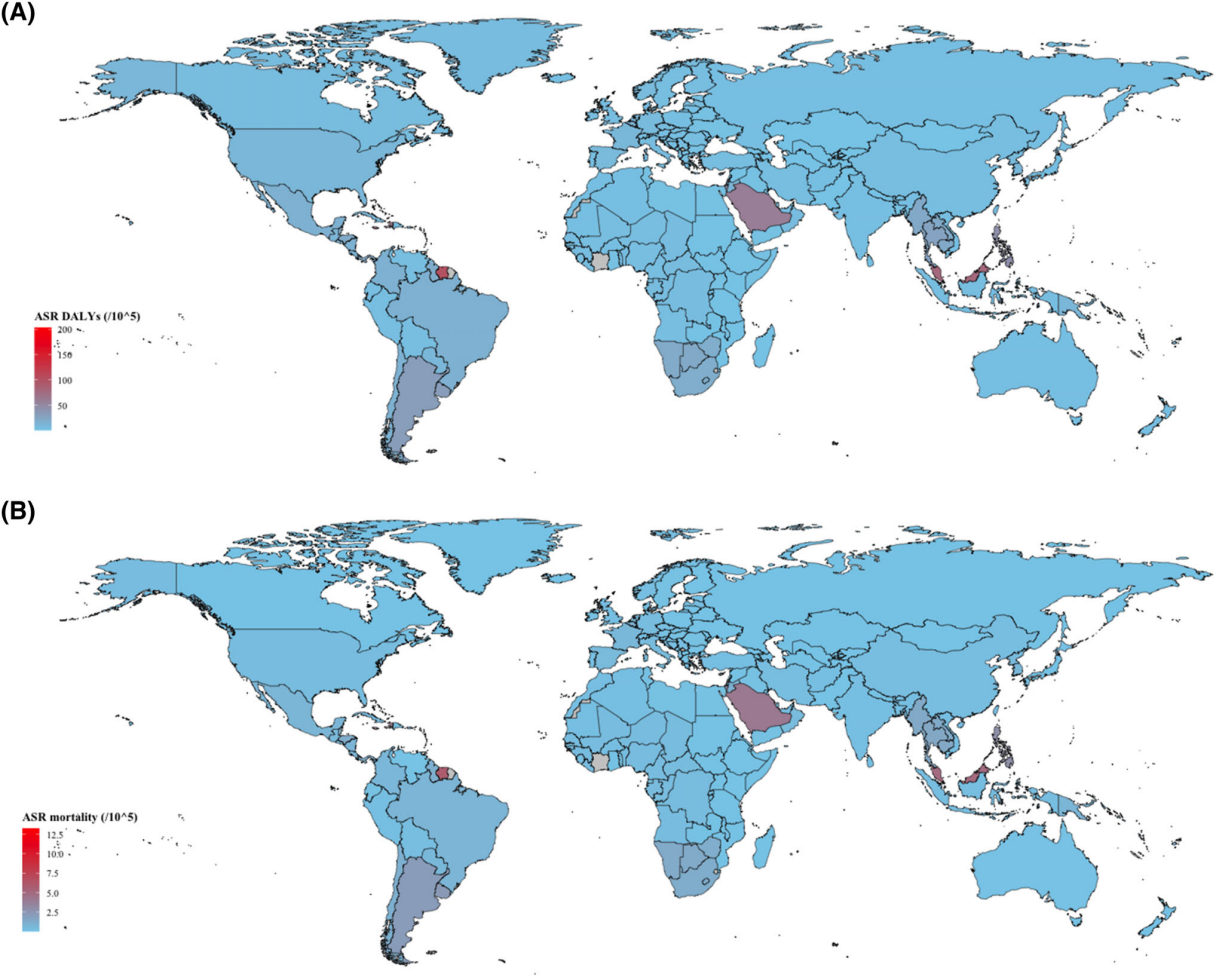
Global age standardized rate (per 100 000) of (A) DALYs and (B) mortality for decubitus ulcer in 2019. DALYs, disability‐adjusted life‐years.

A global map was conducted to illustrate the regional distribution of DU ASR for DALYs and mortality in 2019 (Figure 1). The numbers of DALYs for DU worldwide in 1990 and 2019 were estimated at 267845.79 (95% UI: 211023.57–360562.28) and 481422.76 (95% UI: 374333.94–583429.14) respectively. The global ASRs (per 100 000) of DU DALYs increased from 7.78 (95% UI: 6.16–10.41) in 1990 to 6.23 (95% UI: 4.83–7.55) in 2019. Globally, there was a decreasing trend observed in the DALYs burden of DU with the EAPC of −0.82 (95% UI: −0.93 to −0.72). As shown in Table 1, the regions that have experienced an increase in DALYs from 1990 to 2019 include Central Europe, Eastern Europe, Southeast Asia, East Asia, Central Asia, Southern Latin America, Western Sub‐Saharan Africa, North Africa and Middle East and Oceania. The regions that have witnessed a decline in DALYs were Western Europe, High‐income Asia Pacific, Australasia, High‐income North America, Central Latin America, Andean Latin America, Eastern Sub‐Saharan Africa, Southern Sub‐Saharan Africa, Central Sub‐Saharan Africa, Caribbean.

**TABLE 1:** The number and age‐standardized rate of DALYs for decubitus ulcer globally and by GBD region in 1990 and 2019.

Location	1990		2019		EAPC (95% CI)
	Number (95% UI)	ASR (95% UI), per 100 000	Number (95% UI)	ASR (95% UI), per 100 000	
Global	267845.79 (211023.57–360562.28)	7.78 (6.16–10.41)	481422.76 (374333.94–583429.14)	6.23 (4.83–7.55)	−0.82 (−0.93 to −0.72)
GBD region					
Central Europe	3285.24 (2521.31–4627.11)	2.43 (1.88–3.38)	6516.09 (4800.15–8169.34)	3.20 (2.33–3.99)	1.71 (1.41 to 2.00)
Eastern Europe	5438.37 (3803.24–10510.37)	2.08 (1.47–3.87)	7851.64 (5605.77–11122.75)	2.53 (1.75–3.62)	0.88 (0.74 to 1.02)
Western Europe	66514.44 (47775.02–96217.29)	11.39 (8.2–16.24)	71452.35 (52142.43–97252.94)	6.87 (5.10–9.43)	−2.24 (−2.43 to −2.06)
Southeast Asia	31964.91 (25557.20–43939.88)	13.04 (10.42–18.31)	95781.14 (71204.49–114920.00)	17.84 (13.45–21.34)	1.37 (1.10 to 1.64)
East Asia	17413.62 (14096.99–25650.59)	2.78 (2.27–4.02)	57567.13 (46654.24–69206.72)	3.54 (2.86–4.21)	1.13 (0.13 to 2.14)
Central Asia	238.00 (179.65–335.24)	0.53 (0.40–0.75)	361.69 (264.61–463.64)	0.60 (0.43–0.76)	0.68 (0.57 to 0.80)
South Asia	9098.55 (3620.86–12944.40)	2.04 (0.81–2.97)	27548.92 (13038.07–36089.80)	2.34 (1.14–3.03)	0.13 (−0.08 to 0.35)
High‐income Asia Pacific	10109.46 (8229.16–12864.11)	5.73 (4.61–7.24)	17911.05 (14315.83–22086.98)	3.80 (3.02–4.74)	−1.54 (−1.67 to −1.41)
Australasia	1159.09 (870.04–1770.18)	5.25 (3.98–7.99)	1417.51 (1041.92–1969.11)	2.74 (2.02–3.75)	−3.00 (−3.35 to −2.64)
Southern Latin America	8541.22 (5793.34–19803.09)	19.90 (13.55–46.41)	22493.90 (14677.62–32468.18)	26.30 (17.11–38.20)	1.50 (1.02 to 1.98)
Tropical Latin America	14986.11 (10082.14–25991.99)	16.70 (11.45–27.58)	34067.53 (16729.31–43996.25)	14.66 (7.20–18.84)	−0.16 (−0.36 to 0.03)
High‐income North America	48734.85 (35530.84–67596.23)	13.51 (9.91–18.98)	55845.22 (42522.72–77319.02)	8.85 (6.74–12.21)	−1.65 (−1.80 to −1.50)
Central Latin America	22442.20 (14432.93–32883.05)	25.15 (16.67–36.42)	31267.18 (23146.84–48745.15)	13.55 (10.00–21.29)	−2.33 (−2.45 to −2.20)
Andean Latin America	2173.95 (614.99–3155.58)	10.25 (3.17–14.57)	1766.94 (1379.13–2289.78)	3.23 (2.52–4.19)	−5.90 (−6.92 to −4.87)
Western Sub‐Saharan Africa	1608.99 (1133.50–2026.51)	2.14 (1.44–2.68)	5212.08 (3326.68–6510.18)	3.45 (2.04–4.30)	2.36 (2.10 to 2.62)
North Africa and Middle East	6028.90 (4464.38–9628.12)	4.14 (3.01–6.72)	16104.71 (13051.05–19438.51)	4.24 (3.43–5.13)	0.43 (0.26 to 0.61)
Eastern Sub‐Saharan Africa	1353.49 (912.15–1807.56)	1.47 (1.03–1.85)	2414.55 (1806.54–3128.78)	1.23 (0.92–1.60)	−0.79 (−0.89 to −0.68)
Southern Sub‐Saharan Africa	5761.93 (4053.42–6972.09)	22.47 (15.57–27.30)	8805.21 (7132.96–11616.77)	18.34 (14.85–23.7)	−1.03 (−1.47 to −0.59)
Central Sub‐Saharan Africa	1774.19 (663.80–2742.48)	8.83 (3.35–13.48)	2673.67 (1304.55–4216.52)	5.84 (2.75–9.38)	−1.69 (−1.81 to −1.57)
Caribbean	8978.94 (6410.40–11637.04)	35.02 (24.78–44.24)	13718.13 (10030.96–17637.25)	26.79 (19.60–34.45)	−1.03 (−1.15 to −0.91)
Oceania	239.34 (157.47–377.34)	10.32 (6.65–17.49)	646.14 (363.38–982.72)	11.53 (6.66–17.38)	0.54 (0.45 to 0.64)

Abbreviations: ASR, age‐standardized rate; CI, confdence interval; DALYs, disability‐adjusted life‐years; EAPC, estimated annual percentage change; GBD, global burden of disease; UI, uncertainty interval.

In 2019, there were over 24.4 thousand (95% UI: 17.3–31.3) deaths due to DU worldwide, equivalent to a 93.5% rise compared with the number of deaths in 1990 [12.6 thousand (95% UI: 8.9–18.0)]. The global ASRs of deaths for DU increased from 0.44 (95% UI: 0.31–0.63) in 1990 to 0.33 (95% UI: 0.23–0.43) in 2019. A decreasing trend in the death burden of DU was globally observed, with an EAPC of −1.18 (95% UI: −1.35 to −1.01) (Table 2). The regions that have experienced an increase in deaths from 1990 to 2019 include Central Europe, Eastern Europe, Southeast Asia, East Asia, Central Asia, South Asia, Southern Latin America, Western Sub‐Saharan Africa, North Africa and Middle East and Oceania. The regions that have witnessed a decline in deaths are Western Europe, High‐income Asia Pacific, Australasia, Tropical Latin America, High‐income North America, Central Latin America, Andean Latin America, Eastern Sub‐Saharan Africa, Southern Sub‐Saharan Africa, Central Sub‐Saharan Africa, Caribbean.

**TABLE 2:** The number and age‐standardized rate of deaths for decubitus ulcer globally and by GBD region in 1990 and 2019.

Location	1990		2019		EAPC (95% CI)
	Number (95% UI)	ASR (95% UI), per 100 000	Number (95% UI)	ASR (95% UI), per 100 000	
Global	12603.16 (8975.74–17969.62)	0.44 (0.31–0.63)	24388.57 (17299.07–31260.82)	0.33 (0.23–0.43)	−1.18 (−1.35 to −1.01)
GBD region					
Central Europe	69.98 (53.28–140.09)	0.06 (0.04–0.11)	180.13 (93.59–231.02)	0.08 (0.04–0.10)	2.60 (2.00 to 3.19)
Eastern Europe	107.05 (63.01–351.53)	0.05 (0.03–0.14)	185.18 (95.9–339.85)	0.06 (0.03–0.10)	0.96 (0.70 to 1.23)
Western Europe	4204.84 (2575.59–6203.19)	0.73 (0.44–1.08)	4587.75 (2567.14–6785.40)	0.38 (0.21–0.57)	−3.03 (−3.32 to −2.74)
Southeast Asia	1407.58 (1106.28–2003.85)	0.76 (0.60–1.09)	5073.37 (3750.24–6132.78)	1.12 (0.82–1.36)	1.62 (1.37 to 1.87)
Central Asia	7.25 (4.52–13.12)	0.02 (0.01–0.04)	11.05 (5.58–15.38)	0.03 (0.01–0.04)	1.32 (1.14 to 1.50)
East Asia	731.86 (573.14–1175.42)	0.18 (0.14–0.29)	3196.83 (2412.97–3917.46)	0.24 (0.18–0.29)	1.15 (0.03 to 2.28)
South Asia	401.12 (123.78–602.95)	0.13 (0.04–0.19)	1466.77 (604.70–1962.73)	0.15 (0.07–0.21)	0.29 (0.01 to 0.57)
High‐income Asia Pacific	433.07 (292.41–591.88)	0.29 (0.18–0.38)	814.17 (555.72–1128.31)	0.13 (0.09–0.18)	−3.11 (−3.37 to −2.85)
Australasia	54.25 (34.76–100.64)	0.26 (0.17–0.50)	44.44 (23.84–86.41)	0.07 (0.04–0.15)	−5.66 (−6.24 to −5.08)
Southern Latin America	501.70 (329.06–1199.19)	1.28 (0.84–3.09)	1570.12 (942.24–2385.26)	1.81 (1.08–2.74)	1.76 (1.21 to 2.31)
Tropical Latin America	564.30 (348.08–996.14)	0.83 (0.53–1.43)	1645.91 (524.61–2114.52)	0.74 (0.24–0.95)	−0.14 (−0.27 to −0.01)
Central Latin America	842.97 (517.87–1263.4)	1.19 (0.73–1.78)	1320.13 (861.96–2368.38)	0.59 (0.39–1.06)	−2.63 (−2.78 to −2.48)
High‐income North America	1974.08 (1134.67–3028.65)	0.53 (0.31–0.82)	1774.97 (1082.68–3194.24)	0.25 (0.15–0.45)	−2.93 (−3.12 to −2.74)
Andean Latin America	100.33 (25.18–146.86)	0.59 (0.16–0.86)	73.52 (52.17–114.23)	0.14 (0.10–0.22)	−7.31 (−8.61 to −6.00)
Western Sub‐Saharan Africa	58.68 (30.65–78.76)	0.13 (0.07–0.17)	243 (110.53–319.89)	0.23 (0.11–0.31)	2.81 (2.51 to 3.12)
North Africa and Middle East	288.32 (198.33–495.62)	0.26 (0.18–0.46)	792.11 (638.06–993.93)	0.25 (0.20–0.33)	0.26 (0.05 to 0.47)
Southern Sub‐Saharan Africa	302.71 (194.49–378.7)	1.47 (0.93–1.85)	494.83 (381.52–651.20)	1.24 (0.95–1.63)	−0.98 (−1.42 to −0.53)
Eastern Sub‐Saharan Africa	23.00 (12.20–32.38)	0.06 (0.03–0.08)	38.94 (24.97–64.92)	0.04 (0.03–0.07)	−1.33 (−1.47 to −1.19)
Central Sub‐Saharan Africa	68.03 (21.91–107.88)	0.53 (0.18–0.83)	112.20 (44.77–185.9)	0.37 (0.15–0.62)	−1.51 (−1.63 to −1.39)
Caribbean	451.42 (294.09–572.65)	2.05 (1.31–2.61)	732.94 (503.36–965.98)	1.41 (0.97–1.86)	−1.44 (−1.56 to −1.32)
Oceania	10.62 (6.53–18.31)	0.73 (0.41–1.52)	30.20 (16.36–46.60)	0.78 (0.42–1.23)	0.36 (0.28 to 0.45)

Abbreviations: ASR, age‐standardized rate; CI, confdence interval; EAPC, estimated annual percentage change; GBD, global burden of disease; UI, uncertainty interval.

### 3.2 Global burden of DU by age and sex

**FIGURE 2:**
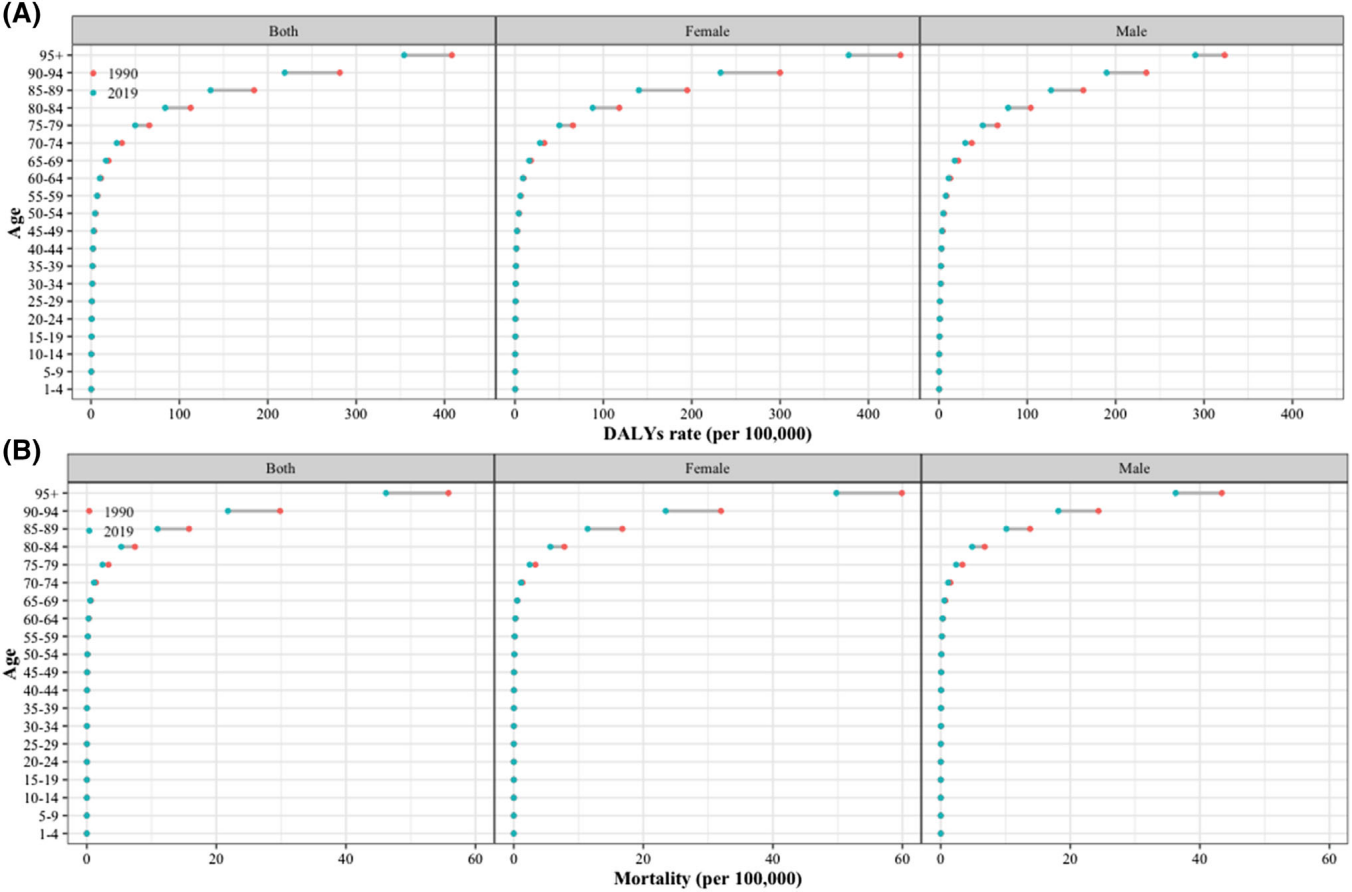
Global rate (per 100 000) of (A) DALYs and (B) mortality for decubitus ulcer by sex from 1990 to 2019. DALYs, disability‐adjusted life‐years.

**FIGURE 3:**
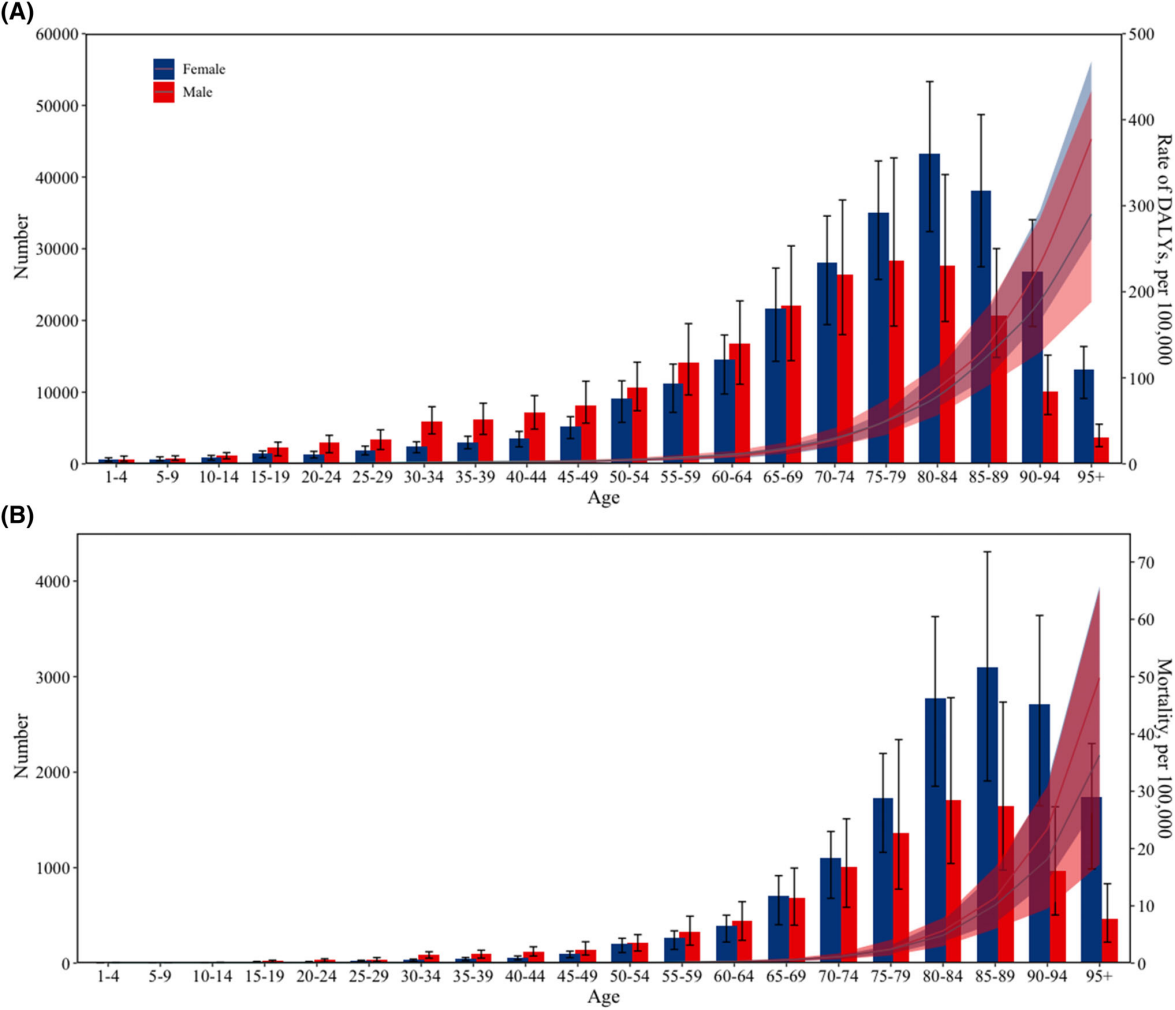
Global number and rate (per 100 000) of (A) DALYs and (B) mortality for decubitus ulcer by sex in 2019. DALYs, disability‐adjusted life‐years.

**FIGURE 4:**
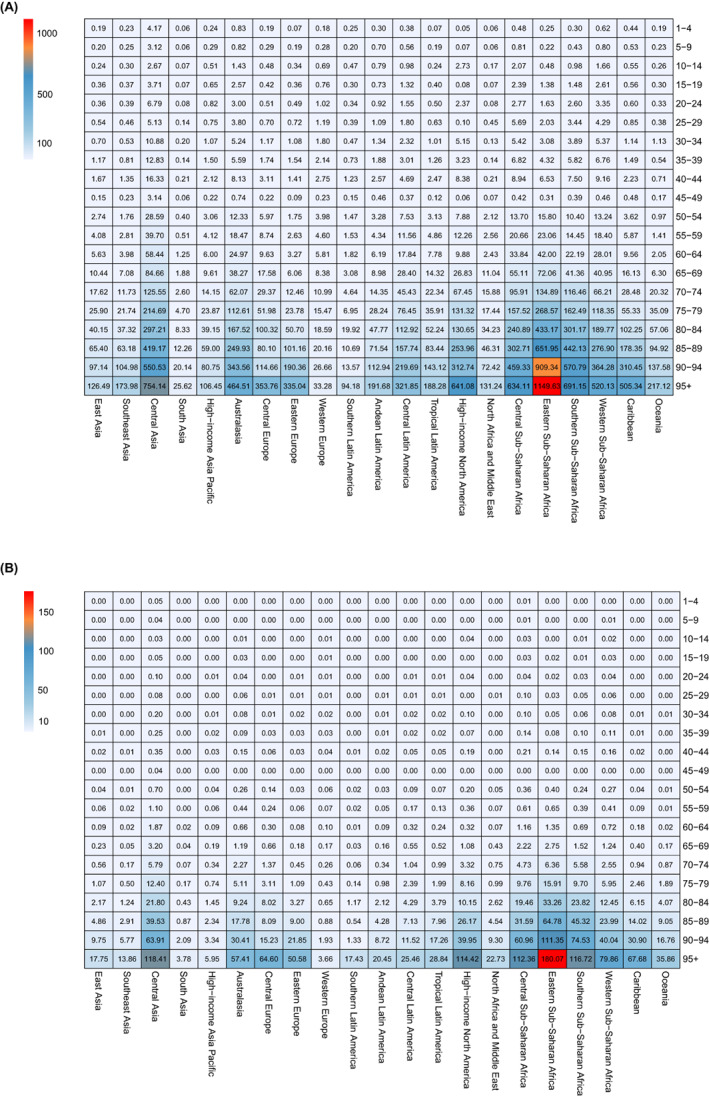
The rate of (A) DALYs and (B) deaths for decubitus ulcer across global regions by age in 2019. DALYs, disability‐adjusted life‐years.

In 2019, the ASR of DALYs for DU was higher in males, whereas the ASR of deaths was higher in females (Table 3). The gender ratio was highest in Western Sub‐Saharan Africa, both in terms of DALYs and mortality rates. The analysis of changes in DALYs and mortality across different age groups from 1990 to 2019 reveals a clear trend: as the population ages, there is a notable escalation in both DALYs and mortality rates (Figure 2). This pattern is particularly pronounced among older aged 75 years and above, irrespective of gender. In 2019, the global DALYs rate increased with age for both men and women. However, it gradually decreased in the elderly population (aged over 85 years) (Figure 3). Furthermore, prior to the age of 70, women had a higher DALYs count compared to men; however, after the age of 70, it became lower than that of men. Regardless of gender, the global death rate increased with age for both men and women in 2019. The number of global deaths increased with age but decreased among individuals over 90 years old. Additionally, before the age of 65, more women died compared to men; however, after the age of 70, this trend reversed. The burden of DALYs and deaths increases significantly with the ageing population worldwide, regardless of the geographical region, particularly among the elderly population in Eastern Sub‐Saharan Africa (Figure 4).

**TABLE 3:** The age‐standardized rates (per 100 000) of DALYs and deaths for decubitus ulcer in male and female population and the gender ratio globally and by GBD region in 2019.

Location	DALYs			Deaths		
	Male (95% UI)	Female (95% UI)	Gender ratio	Male (95% UI)	Female (95% UI)	Gender ratio
Global	6.34 (4.48–8.89)	6.01 (4.44–7.14)	1.05	0.31 (0.19–0.49)	0.34 (0.22–0.44)	0.91
SDI						
High SDI	7.27 (4.84–11.54)	6.69 (5.05–8.50)	1.09	0.27 (0.10–0.59)	0.27 (0.16–0.43)	1.00
High‐middle SDI	6.65 (4.26–9.21)	6.05 (3.89–7.16)	1.10	0.36 (0.19–0.55)	0.38 (0.21–0.47)	0.95
Middle SDI	6.87 (5.46–9.31)	6.84 (4.95–8.16)	1.00	0.36 (0.27–0.53)	0.45 (0.31–0.56)	0.80
Low‐middle SDI	4.69 (2.59–5.64)	4.48 (2.63–5.44)	1.05	0.26 (0.12–0.32)	0.29 (0.15–0.36)	0.90
Low SDI	3.50 (2.18–4.39)	3.43 (2.03–4.55)	1.02	0.20 (0.10–0.26)	0.21 (0.11–0.28)	0.95
GBD region						
Eastern Europe	3.27 (1.94–5.18)	1.97 (1.31–2.57)	1.66	0.06 (0.01–0.17)	0.05 (0.02–0.08)	1.20
Central Europe	3.40 (2.30–4.48)	2.93 (2.11–3.62)	1.16	0.07 (0.03–0.11)	0.09 (0.04–0.11)	0.78
Western Europe	6.28 (3.33–11.10)	7.15 (4.95–9.56)	0.88	0.34 (0.10–0.70)	0.40 (0.20–0.60)	0.85
East Asia	4.43 (3.57–5.74)	2.91 (2.13–3.56)	1.52	0.28 (0.21–0.39)	0.21 (0.14–0.27)	1.33
High‐income Asia Pacific	4.34 (3.33–5.75)	3.21 (2.53–3.95)	1.35	0.13 (0.07–0.22)	0.12 (0.08–0.17)	1.08
Central Asia	0.69 (0.46–1.00)	0.52 (0.34–0.67)	1.33	0.02 (0.01–0.04)	0.03 (0.01–0.04)	0.67
Australasia	2.83 (1.90–4.48)	2.66 (1.98–3.51)	1.06	0.07 (0.02–0.21)	0.08 (0.03–0.12)	0.88
Southeast Asia	17.66 (12.05–21.74)	17.52 (10.46–21.55)	1.01	1.00 (0.70–1.27)	1.19 (0.71–1.46)	0.84
South Asia	1.96 (0.73–2.61)	2.70 (1.16–3.73)	0.73	0.13 (0.04–0.18)	0.17 (0.06–0.25)	0.76
Andean Latin America	3.77 (2.78–4.77)	2.71 (1.96–4.46)	1.39	0.16 (0.09–0.21)	0.13 (0.08–0.26)	1.23
Tropical Latin America	15.87 (5.98–23.39)	13.2 (5.76–15.63)	1.20	0.63 (0.08–0.98)	0.79 (0.22–0.97)	0.80
High‐income North America	9.50 (6.19–15.08)	8.29 (6.21–10.39)	1.15	0.26 (0.07–0.69)	0.24 (0.13–0.42)	1.08
Central Latin America	14.13 (7.91–28.56)	12.89 (8.97–18.63)	1.10	0.54 (0.19–1.36)	0.63 (0.39–1.11)	0.86
Southern Latin America	25.20 (9.40–44.43)	26.30 (14.91–38.75)	0.96	1.59 (0.51–2.92)	1.90 (1.04–3.01)	0.84
Western Sub‐Saharan Africa	5.14 (2.45–6.62)	1.96 (1.24–2.70)	2.62	0.36 (0.12–0.48)	0.12 (0.06–0.19)	3.00
Eastern Sub‐Saharan Africa	1.24 (0.90–1.62)	1.17 (0.84–1.66)	1.06	0.02 (0.02–0.04)	0.05 (0.03–0.10)	0.40
North Africa and Middle East	4.34 (3.21–5.41)	4.12 (3.14–5.27)	1.05	0.25 (0.19–0.34)	0.26 (0.18–0.34)	0.96
Central Sub‐Saharan Africa	5.53 (2.56–8.72)	5.89 (1.61–11.46)	0.94	0.31 (0.11–0.49)	0.40 (0.09–0.78)	0.78
Southern Sub‐Saharan Africa	15.95 (13.15–23.68)	19.06 (13.75–25.90)	0.84	0.93 (0.75–1.44)	1.37 (0.96–1.89)	0.68
Caribbean	24.96 (15.46–35.88)	27.86 (20.40–35.89)	0.90	1.13 (0.59–1.78)	1.60 (1.07–2.10)	0.71
Oceania	6.53 (3.33–10.92)	15.86 (8.27–24.73)	0.41	0.38 (0.19–0.65)	1.07 (0.53–1.72)	0.36

Abbreviations: DALYs, disability‐adjusted life‐years; GBD, global burden of disease; SDI, sociodemographic index; UI, uncertainty interval.

### 3.3 Global trend of DU by SDI

**FIGURE 5:**
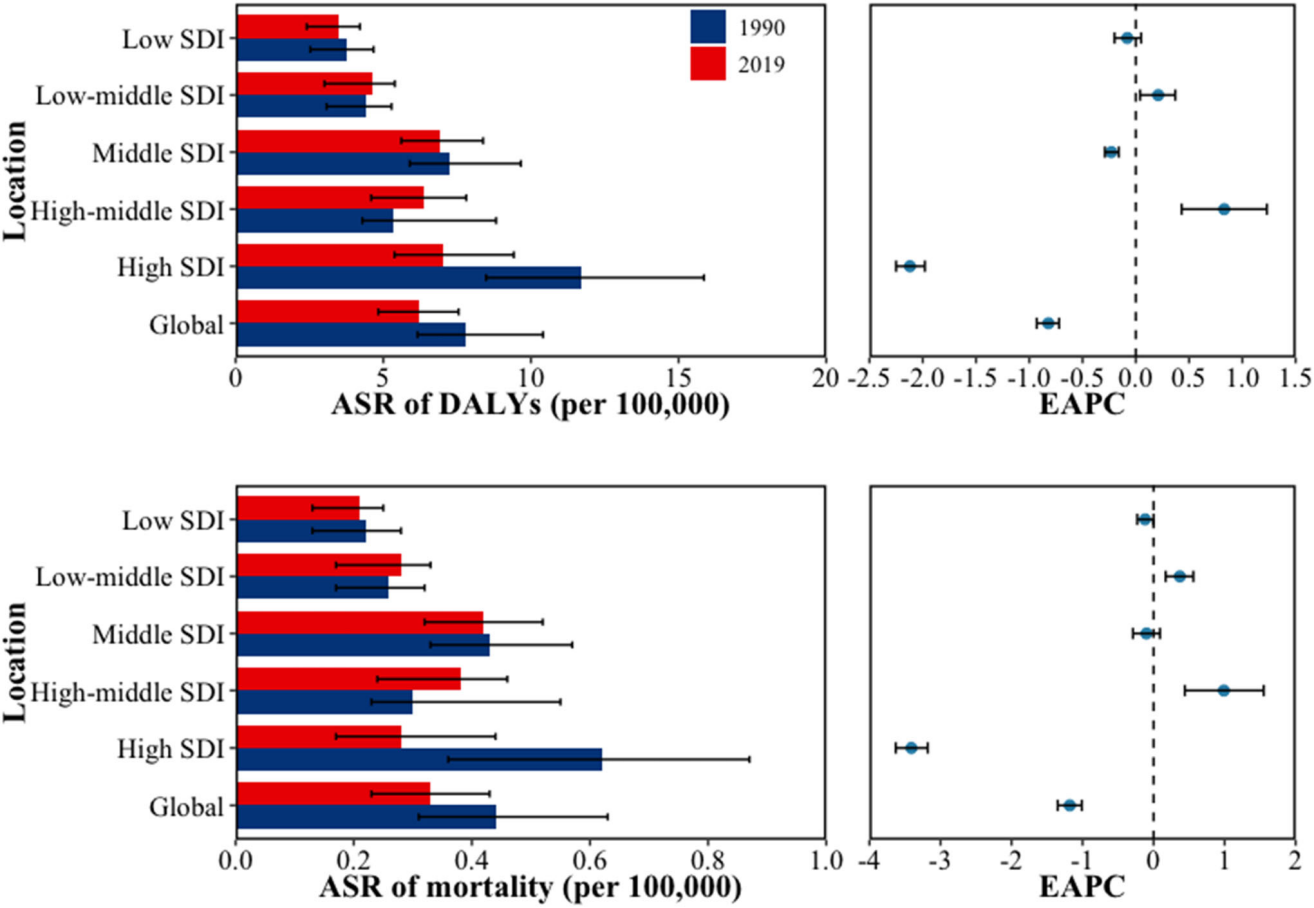
The Age‐standardized rate and changes of DALYs and mortality due to decubitus ulcer by SDI from 1990 to 2019. ASR, age‐standardized rate; DALYs, disability‐adjusted life‐years; SDI, sociodemographic index.

**FIGURE 6:**
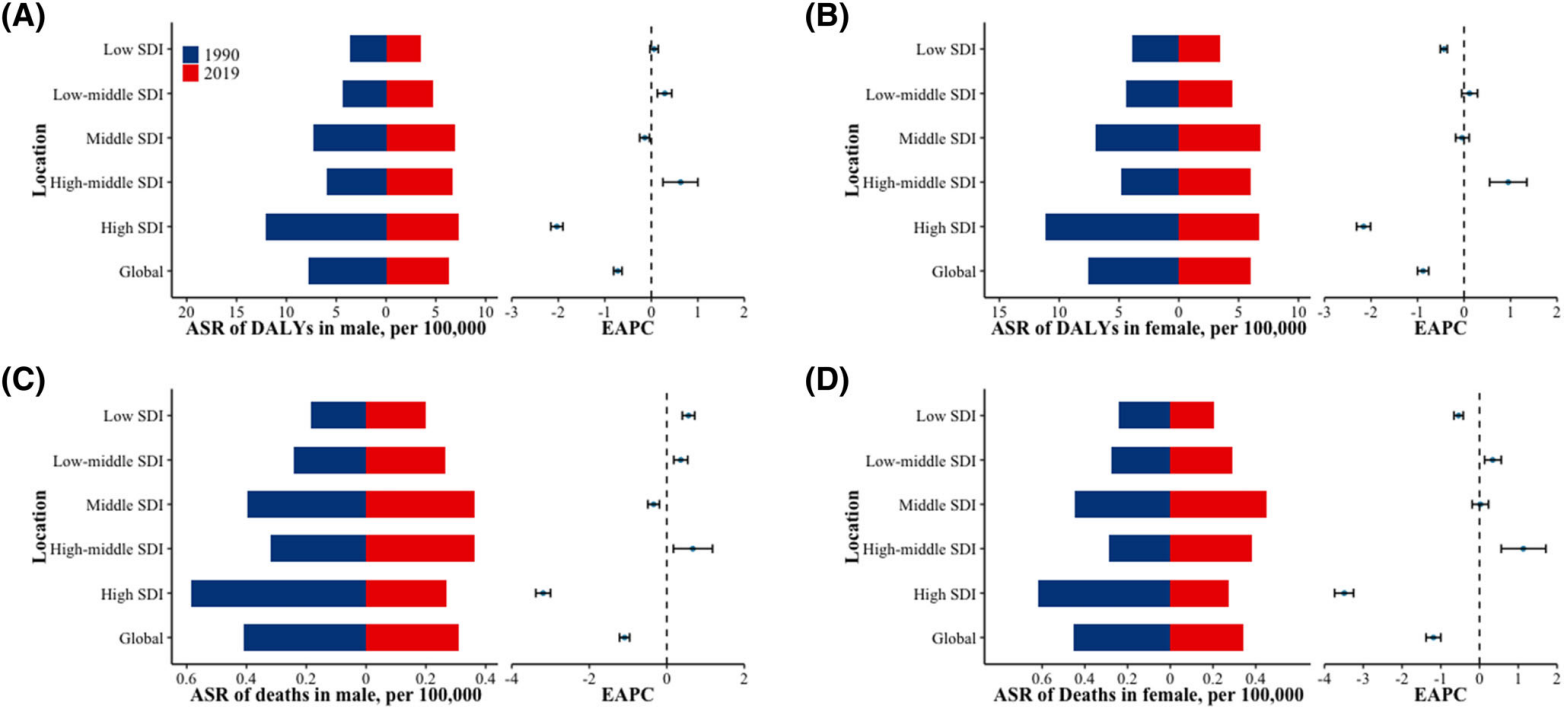
The Age‐standardized rate of DALYs and mortality due to decubitus ulcer by SDI in male and female from 1990 to 2019. (A) the ASR of DALYs in male by SDI. (B) the ASR of DALYs in female by SDI. (C) the ASR of eaths in male by SDI. (D) the ASR of deaths in female by SDI. ASR, age‐standardized rate; DALYs, disability‐adjusted life‐years; SDI, sociodemographic index.

**FIGURE 7:**
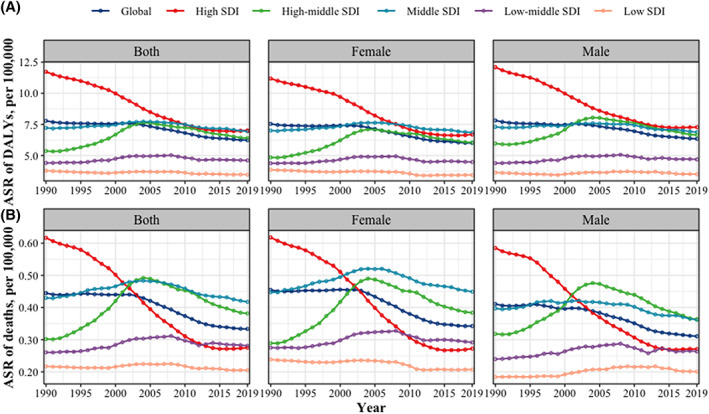
The trend of Age‐standardized rate for (A) DALYs and (B) mortality due to decubitus ulcer by sex and SDI from 1990 to 2019. ASR, age‐standardized rate; DALYs, disability‐adjusted life‐years; SDI, sociodemographic index.

In terms of the ASR and changes in DALYs and mortality from 1990 to 2019, countries with high SDI exhibited the most significant decline (EAPC and its 95% CI smaller than 0), while those with high‐middle SDI experienced the greatest increase (EAPC and its 95% CI >0) (Figure 5). The DALYs for both males and females decreased in areas with high SDI (EAPC and its 95% CI smaller than 0), while they increased in areas with medium‐high SDI (EAPC and its 95% CI >0). However, there were gender‐specific trends observed in middle, low‐middle and low SDI areas. Men showed no change trend in low SDI areas (EAPC and its 95% CI contain 0) while women exhibited a downward trend (EAPC and its 95% CI smaller than 0). In low‐middle SDI areas, men experienced an increase while women remained unchanged (EAPC and its 95% CI contain 0). In the middle SDI region, there was a decrease (EAPC and its 95% CI smaller than 0) among males with no change observed among females. With regards to mortality rates, both men and women experienced a decrease (EAPC and its 95% CI smaller than 0) in high SDI areas, while high‐middle and low‐middle SDI areas saw an increase (EAPC and its 95% CI greater than 0). The change trends in the middle, low‐middle and low SDI areas exhibit divergences between men and women. In the latter area, male mortality has increased (EAPC and its 95% CI greater than 0) while female mortality has decreased (EAPC and its 95% CI smaller than 0); whereas in the former area, male mortality has decreased (EAPC and its 95% CI smaller than 0) but female mortality remains unchanged (EAPC and its 95% CI contain 0) (Figure 6). After further stratifying by gender within the SDI group, we observed a consistent pattern in DALYs and deaths for both genders combined. However, there was a disparity in mortality rates between the high‐middle SDI and middle SDI groups from 2000 to 2015. During this period, the mortality rate of both high‐middle and middle SDI groups remained relatively similar overall; however, among females, the middle SDI group exhibited a higher mortality rate compared to the high‐middle SDI group. Conversely, among males, this trend was reversed (Figure 7).

## 4 DISCUSSION

The management of chronic wounds poses a formidable challenge for all nations worldwide, encompassing both clinical medicine and public health perspectives. The cost of DU care is substantial, and a study utilized economic modelling techniques to evaluate the financial burden associated with hospital‐acquired pressure injury (HAPI), revealing that HAPI expenses can surpass $26.8 billion annually in the United States.^9^ In addition to these direct costs (including wound dressings, biologics and grafts), wounds incur substantial expenses related to medical care and surgical procedures, as well as productivity loss due to impaired labor.^3^ Assessing the medical costs linked to wound healing accurately is often challenging due to various factors. Most current assessment protocols fail to consider secondary costs, such as those arising from complications resulting from chronic wounds (e.g. amputation, sepsis), which may be difficult to attribute directly to the aetiology of the wound itself. Furthermore, the complexity of wound care is compounded by its provision across different levels of healthcare facilities ranging from community hospitals to general hospitals.

According to the updated 2019 International Guidelines for the Prevention and Treatment of Pressure Ulcers/Injuries, pressure ulcers/pressure injuries are defined as localized damage to the skin and/or underlying tissues resulting from either pressure alone or a combination of pressure and shear injuries.^10^ Pressure injuries can affect individuals of all ages and health conditions, but they are particularly prevalent among older adults and critically ill patients with multiple comorbidities.^11^, ^12^ It is often regarded as a measure of the quality of care provided.^13^ Despite substantial investments in prevention and treatment efforts, pressure injuries remain prevalent due to factors such as the ageing population, an increasing prevalence of predisposing factors like obesity, diabetes and cardiovascular diseases, as well as limited understanding regarding their treatment mechanisms and preventive measures. Incidence rates vary across different clinical settings: reaching up to 60% in patients with quadriplegia or spinal cord injury, up to 66% in those undergoing prolonged surgeries, and up to 70% in elderly patients with hip or femur fractures.^3^

The development of an accurate disease burden model has the potential to facilitate rational allocation of medical resources and enhance healthcare at various levels. The present study represents the first comprehensive analysis of global DU mortality and DALYs from 1990 to 2019 across more than 200 countries and regions. Our findings demonstrate a decline in age‐standardized DALYs and percentage changes in mortality between 1990 and 2019, indicating a reduction in the global burden of DU as observed in the GBD 2019 study, which implies that significant advancements have been made in the management of DU and associated complications over a span of 30 years, owing to continuous progress in negative‐pressure technology, diverse wound dressing options, strategies for antibiotics and nutritional support, innovative concepts in wound management, as well as ongoing promotion of protocols for DU prevention and treatment.^14^ However, it is worth noting that certain countries and regions have exhibited a concerning upward trend in DALYs and mortality rates. Our findings indicate that Central Europe, Southeast Asia and Western Sub‐Saharan Africa experienced the most significant increases between 1990 and 2019. A systematic review study revealed that the prevalence of DU in Europe was estimated to be 10.8% (ranging from 4.6% to 27.2%). According to the GBD 2017 study, the prevalence of DU in EU15+ countries has shown an upward trend from 1990 to 2017, with Denmark and Germany bearing the highest burden of DU.^15^ In 2019, the elderly population over the age of 65 in the European Union's member states reached a staggering figure of approximately 90.5 million individuals, accounting for around one‐fifth (20.3%) of the total population—a demographic shift towards an ‘ageing society’(https://ec.europa.eu/eurostat/). Consequently, there has been a corresponding increase in disabled elderly individuals as well as DU‐associated DALYs within this region. On another note, it should be acknowledged that Europe boasts a relatively robust healthcare system which provides patients with DU access to high‐quality medical care and prolonged treatment options aimed at maintaining their quality of life; however, this may also contribute to an increase in DALYs. In the past decade, South‐East Asia has witnessed significant socio‐economic development and population growth, resulting in lifestyle changes that have contributed to the escalating prevalence of chronic non‐communicable diseases, which account for 60% of deaths in the region. Due to the relatively weak health systems and uneven financial resources, many Southeast Asian countries are unable to learn from interventions implemented in countries with more advanced healthcare systems.^16^, ^17^ The prioritization of prevention, management and treatment in these specific areas is imperative for public health policymakers.

The SDI serves as a comprehensive indicator reflecting the developmental status of a country or region.^5^ Regions with high SDI typically exhibit more advanced healthcare systems, characterized by long‐term research efforts in chronic wound treatment and prevention, leading to the establishment of standardized treatment protocols. However, in areas with high‐middle SDI levels, disparities may arise in the development of medical services despite improvements in population size and living standards, consequently resulting in an increase in DALYs and mortality rates. The lower prevalence of DU in low SDI countries can be attributed to their comparatively shorter life expectancy and lower rates of population ageing. Meanwhile, the inadequate provision of healthcare in regions characterized by low (SDI) and the absence of robust health information systems can contribute to underreporting and underdetection of DU. The relationship between a country's level of development and the prevalence of DU is still not clearly defined. Regarding gender, most regions worldwide exhibit higher DALY rates for men and higher mortality rates for women. However, reports on the association between gender and DU have been inconsistent. Some studies suggest that gender cannot be considered an independent risk factor for developing DU, as there are no statistically significant differences in their prevalence and incidence between men and women.^18^ Nevertheless, certain studies have observed a potentially higher prevalence of DU among men compared to women, possibly due to a higher prevalence of smoking among men.^19^ Discrepancies in analysis may primarily stem from variations in databases used and analytical methods employed. In terms of age, regardless of gender, there is a positive correlation between increasing age and higher DALYs and mortality rates associated with DU disease. This finding aligns with the conclusions drawn from relevant reviews and meta‐analyses on DU.^3^, ^11^ Consequently, it is imperative to develop targeted public health policies addressing DU in the elderly population. Although young individuals exhibit lower DALYs and mortality rates attributed to DU compared to middle‐aged and elderly individuals, it is crucial not to overlook the occurrence of DU among young people resulting from spinal cord injuries or other causes. Notably, one study highlighted that approximately one‐third of patients with spinal cord injuries suffer from DU, with Africa being particularly affected.^20^

The COVID‐19 pandemic has significantly disrupted global healthcare, including wound care. Factors associated with a poor prognosis for COVID‐19 mirror those that elevate the risk of chronic wounds, encompassing conditions such as diabetes, hypertension, obesity and renal dysfunction.^12^ These underlying risk factors greatly heighten the susceptibility of individuals with COVID‐19 to the development of DU and experiencing severe consequences like infection, sepsis, amputation or even mortality.^12^, ^21^ There are two primary categories of pressure injuries induced by COVID‐19: those caused by protective devices during preventive measures and those resulting from prolonged prone positions during treatment.^22^ The increased risk of DU in individuals infected with COVID‐19 may be attributed to DU occurring in typical anatomical regions due to limited mobility and prolonged exposure to the same position during treatment. Simultaneously, alterations in the body's immune metabolism can impact wound healing progress, as well as abnormal inflammatory and coagulation responses following infection.^23^, ^24^ Additionally, it is noteworthy that COVID‐19, being a global pandemic, could potentially exert a greater strain on healthcare systems within ageing countries and those with low SDI, leading to an escalation in the burden of chronic diseases; however, there is currently insufficient updated data available to substantiate this perspective.

In the management of DU, prevention should take precedence over treatment. To enhance public awareness of the disease, its risk factors, consequences and treatment strategies, it is imperative to promote health education extensively. While various methods can potentially alleviate the burden caused by DU; however, due to regional cultural disparities and national circumstances, progress in their management remains uneven.^3^, ^11^, ^15^ Therefore, based on the aforementioned data, we hope that public health policymakers will formulate more targeted and tailored health policies while rationalizing the allocation of medical resources worldwide to reduce the global disease burden of DU.

## 5 LIMITATIONS

This study has several limitations to consider. First, the results of GBD study estimates rely on the quality and quantity of data incorporated into the GBD study. Second, the GBD estimates do not reflect the situation in countries with inadequate monitoring systems, as the GBD study is based on a national level. Third, we are unable to explore the relevant factors affecting the development of DU due to the accessibility of the GBD data currently available. Furthermore, the Global Burden of Disease database has not been updated to incorporate data beyond 2019, thereby restricting our access to information post‐2019. Despite potential fluctuations induced by the Covid‐19 pandemic, our analysis indicates a downward trend in the global burden of DU over time. We maintain confidence that our scientific analysis can still yield valuable insights into the current global prevalence of DU.

## 6 CONCLUSION

Globally, although the number of DU DALYs and death cases increased between 1990 and 2019, the ASRs of DU DALYs and mortality exhibited a downward trend. This suggests that the burden of DU was perceived to be decreasing in most regions. However, there was an escalation in the burden of DU among older age groups (both men and women) and in countries with high‐middle SDI. Strengthening population‐based data on DU prevalence and implementing dynamic monitoring of the burden of this condition at the public health level will enable policymakers to develop more evidence‐based and rational strategies for allocating healthcare resources.

## CONFLICT OF INTEREST STATEMENT

The authors declare that there are no potential conflicts interest relevant to this article.

## Data Availability

The data that support the findings of this study are openly available, if it is accepted for publication.

## References

[1] Huang Y , Mao B , Ni P , et al. Investigation on the status and determinants of caregiver burden on caring for patients with chronic wound. Adv Wound Care. 2019;8:429‐437. 10.1089/wound.2018.0873 PMC6703242 31440420

[2] Alam W , Hasson J , Reed M . Clinical approach to chronic wound management in older adults. J Am Geriatr Soc. 2021;69:2327‐2334. 34002364 10.1111/jgs.17177

[3] Hajhosseini B , Longaker MT , Gurtner GC . Pressure injury. Ann Surg. 2020;271:671‐679. 31460882 10.1097/SLA.0000000000003567

[4] Global burden of 369 diseases and injuries in 204 countries and territories, 1990‐2019: a systematic analysis for the global burden of disease study 2019. Lancet. 2020;396:1204‐1222. 33069326 10.1016/S0140-6736(20)30925-9 PMC7567026

[5] Quantifying risks and interventions that have affected the burden of diarrhoea among children younger than 5 years: an analysis of the global burden of disease study 2017. Lancet Infect dis. 2020;20:37‐59. 31678029 10.1016/S1473-3099(19)30401-3 PMC7340495

[6] Zhang S , Cheng C , Lin Z , et al. The global burden and associated factors of ovarian cancer in 1990‐2019: findings from the global burden of disease study 2019. BMC Public Health. 2022;22:1455. 35907822 10.1186/s12889-022-13861-y PMC9339194

[7] Global age‐sex‐specific fertility, mortality, healthy life expectancy (HALE), and population estimates in 204 countries and territories, 1950–2019: a comprehensive demographic analysis for the global burden of disease study 2019. Lancet. 2020;396:1160‐1203. 33069325 10.1016/S0140-6736(20)30977-6 PMC7566045

[8] Zhang W , Cao G , Wu F , et al. Global burden of prostate cancer and association with socioeconomic status, 1990–2019: a systematic analysis from the global burden of disease study. J Epidemiol Glob HEA. 2023;13:407‐421. 10.1007/s44197-023-00103-6 PMC10469111 37147513

[9] Padula WV , Delarmente BA . The national cost of hospital‐acquired pressure injuries in the United States. Int Wound J. 2019;16:634‐640. 30693644 10.1111/iwj.13071 PMC7948545

[10] Kottner J , Cuddigan J , Carville K , et al. Prevention and treatment of pressure ulcers/injuries: the protocol for the second update of the international clinical practice guideline 2019. J Tissue Viability. 2019;28:51‐58. 30658878 10.1016/j.jtv.2019.01.001

[11] Martinengo L , Olsson M , Bajpai R , et al. Prevalence of chronic wounds in the general population: systematic review and meta‐analysis of observational studies. Ann Epidemiol. 2019;29:8‐15. 30497932 10.1016/j.annepidem.2018.10.005

[12] Sen CK . Human wound and its burden: updated 2020 compendium of estimates. Adv Wound Care. 2021;10:281‐292. 10.1089/wound.2021.0026 PMC8024242 33733885

[13] Palfreyman SJ , Stone PW . A systematic review of economic evaluations assessing interventions aimed at preventing or treating pressure ulcers. Int J Nurs Stud. 2015;52:769‐788. 25012958 10.1016/j.ijnurstu.2014.06.004

[14] Teot L , Ohura N . Challenges and Management in Wound Care. Plast Reconstr Surg. 2021;147:9S‐15S. 33347058 10.1097/PRS.0000000000007628

[15] Goodall R , Armstrong A , Hughes W , et al. Trends in decubitus ulcer disease burden in European Union 15+ countries, from 1990 to 2017. PRS‐Glob Open. 2020;8:e3252. 10.1097/GOX.0000000000003252 PMC7722593 33299715

[16] Fritz M , Fromell H . How to dampen the surge of non‐communicable diseases in Southeast Asia: insights from a systematic review and meta‐analysis. Health Policy Plann. 2022;37:152‐167. 10.1093/heapol/czab138 PMC8757494 34791261

[17] Dans A , Ng N , Varghese C , Tai ES , Firestone R , Bonita R . The rise of chronic non‐communicable diseases in Southeast Asia: time for action. Lancet. 2011;377:680‐689. 21269677 10.1016/S0140-6736(10)61506-1

[18] Lichterfeld‐Kottner A , Lahmann N , Kottner J . Sex‐specific differences in prevention and treatment of institutional‐acquired pressure ulcers in hospitals and nursing homes. J Tissue Viability. 2020;29:204‐210. 32471633 10.1016/j.jtv.2020.05.001

[19] Raeder K , Jachan DE , Muller‐Werdan U , et al. Prevalence and risk factors of chronic wounds in nursing homes in Germany: a cross‐sectional study. Int Wound J. 2020;17:1128‐1134. 32815303 10.1111/iwj.13486 PMC7949346

[20] Shiferaw WS , Akalu TY , Mulugeta H , Aynalem YA . The global burden of pressure ulcers among patients with spinal cord injury: a systematic review and meta‐analysis. BMC Musculoskel Dis. 2020;21:334. 10.1186/s12891-020-03369-0 PMC7260823 32471497

[21] Gefen A , Ousey K . COVID‐19: pressure ulcers, pain and the cytokine storm. J Wound Care. 2020;29:540‐542. 33052791 10.12968/jowc.2020.29.10.540

[22] Yu JN , Wu BB , Feng LP , Chen HL . COVID‐19 related pressure injuries in patients and personnel: a systematic review. J Tissue Viability. 2021;30:283‐290. 33895045 10.1016/j.jtv.2021.04.002 PMC8056785

[23] Menachery VD , Gralinski LE . Coagulation and wound repair during COVID‐19. J Heart Lung Transpl. 2021;40:1076‐1081. 10.1016/j.healun.2021.06.006 PMC8195688 34334300

[24] Hirano T . IL‐6 in inflammation, autoimmunity and cancer. Int Immunol. 2021;33:127‐148. 33337480 10.1093/intimm/dxaa078 PMC7799025

